# Supplementary material for: Automated—Mechanical Procedure Compared to Gentle Enzymatic Tissue Dissociation in Cell Function Studies
Source: Biomolecules. 2022 May 14;12(5):701. doi: 10.3390/biom12050701 (PMC9138555; doi:10.3390/biom12050701)
Supplement: Supplementary file 1 [file biomolecules-12-00701-s001.zip › biomolecules-1685733-SM.pdf]

SUPPLEMENTARY

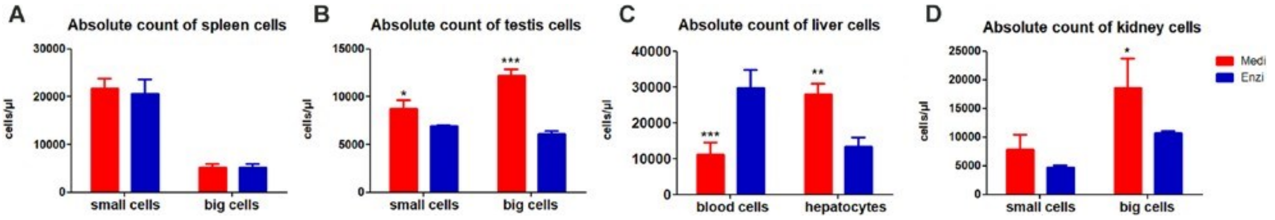

Figure S1. Cell yields obtained from different tissue disaggregation. (A) Statistical histogram of absolute count of spleen cells obtained from Medimachine and enzymatic disaggregation. (B) Statistical histogram of absolute count of testis cells obtained from Medimachine and enzymatic disaggregation. (C) Statistical histogram of absolute count of liver cells obtained from Medimachine and enzymatic disaggregation. (D) Statistical histogram of absolute count of liver cells obtained from Medimachine and enzymatic disaggregation. Two-way ANOVA with Bonferroni's multiple comparison test revealed significant difference (*$p < 0.05$, **$p < 0.01$, ***$p < 0.001$) between the two procedures.

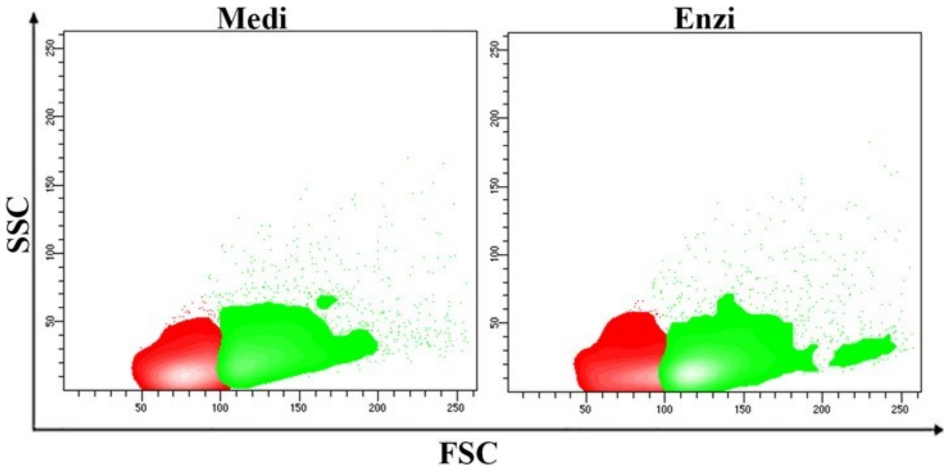

Figure S2. Spleen characteristics Gating cell populations of small cells (red) and big cells (green) in the SSC versus FSC contour plot for the further analysis.

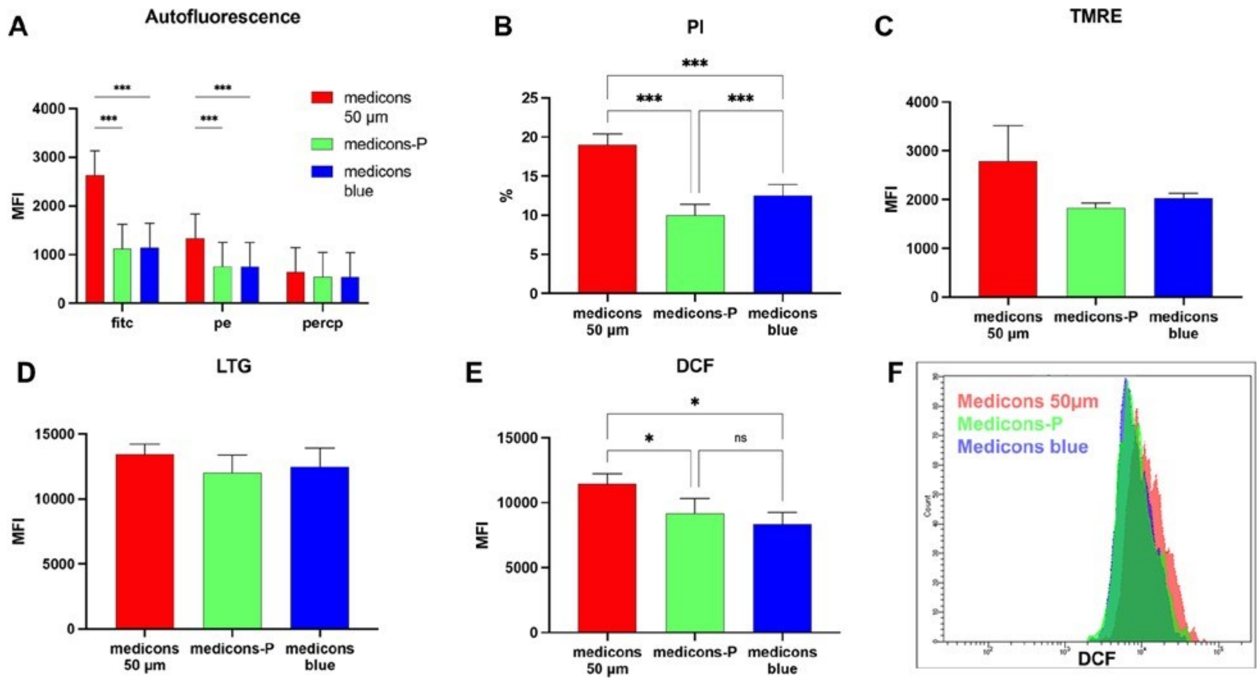

Figure S3. Liver cells by different Medicons approach: Automated-mechanical approach. (A) Statistical histogram of hepatocytes related autofluorescence for three different Medicons in Fitc, PE, Percp fluorescences (***p < 0.001). (B) Statistical histogram of cell surface damage percentage(***p < 0.001). (C) Statistical histogram of mitochondrial membrane potential (Δψ-MMP). (D) Statistical histogram of lysosomal network. (E) Statistical histogram of intracellular ROS presence (H2O2 content). Two-way ANOVA with Bonferroni's multiple comparison test revealed significant difference (*p < 0.05) between different Medicons. (F) Representative cytometric histograms overlay of intracellular ROS presence of Medicons 50µm (red histogram), Medicons-P (green histogram) and Medicons blue (blue histogram).

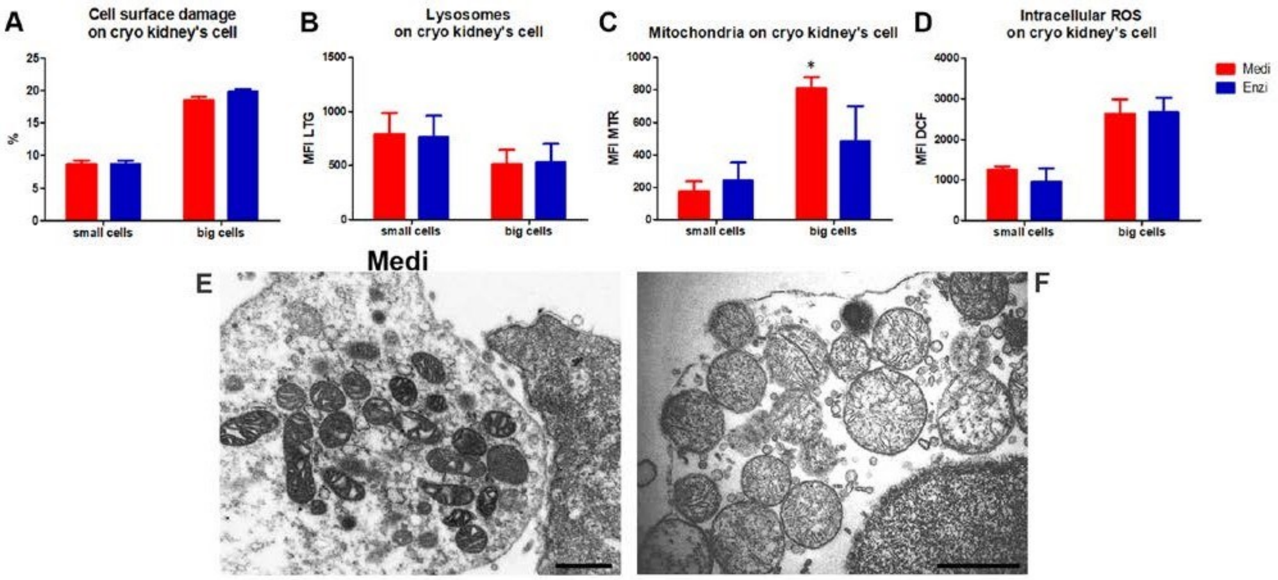

Figure S4. Analyses on cryo cells of the kidney. (A) Statistical histogram of cell surface damage percentage. Two-way ANOVA with Bonferroni's multiple comparison test revealed significant difference between Medimachine II and enzymatic procedure. (B) Statistical histogram of lysosomal network. Two-way ANOVA with Bonferroni's multiple comparison test revealed significant difference between Medimachine II and enzymatic procedure. (C) Statistical histogram of mitochondrial membrane potential (Δψ- MMP). Two-way ANOVA with Bonferroni's multiple comparison test revealed significant difference (*p < 0.05) between Medimachine II and enzymatic procedure. (D) Statistical histogram of intracellular ROS presence (H2O2 content). Two-way ANOVA with Bonferroni's multiple comparison test revealed significant difference between Medimachine II and enzymatic procedure. (E,F) Ultrastructural analyses of cryo cells from kidney both disaggregation methods. Bar = 1µm
